# The *Candida albicans* TOR-Activating GTPases Gtr1 and Rhb1 Coregulate Starvation Responses and Biofilm Formation

**DOI:** 10.1128/mSphere.00477-17

**Published:** 2017-11-15

**Authors:** Peter R. Flanagan, Ning-Ning Liu, Darren J. Fitzpatrick, Karsten Hokamp, Julia R. Köhler, Gary P. Moran

**Affiliations:** a Division of Oral Biosciences, Dublin Dental University Hospital, University of Dublin, Trinity College Dublin, Dublin, Ireland; b Boston Children’s Hospital and Harvard Medical School, Boston, Massachusetts, USA; c School of Genetics and Microbiology, University of Dublin, Trinity College Dublin, Dublin, Ireland; University of Texas Health Science Center

**Keywords:** *Candida albicans*, TOR, biofilm, virulence

## Abstract

Target of rapamycin complex 1 (TORC1) is an essential regulator of metabolism in eukaryotic cells and in the fungal pathogen *Candida albicans* regulates morphogenesis and nitrogen acquisition. Gtr1 encodes a highly conserved GTPase that in *Saccharomyces cerevisiae* regulates nitrogen sensing and TORC1 activation. Here, we characterize the role of *C. albicans GTR1* in TORC1 activation and compare it with the previously characterized GTPase Rhb1. A homozygous *gtr1/gtr1* mutant exhibited impaired TORC1-mediated phosphorylation of ribosomal protein S6 and increased susceptibility to rapamycin. Overexpression of *GTR1* impaired nitrogen starvation-induced filamentous growth, *MEP2* expression, and growth in bovine serum albumin as the sole nitrogen source. Both *GTR1* and *RHB1* were shown to regulate genes involved in ribosome biogenesis, amino acid biosynthesis, and expression of biofilm growth-induced genes. The *rhb1/rhb1* mutant exhibited a different pattern of expression of Sko1-regulated genes and increased susceptibility to Congo red and calcofluor white. The homozygous *gtr1/gtr1* mutant exhibited enhanced flocculation phenotypes and, similar to the *rhb1/rhb1* mutant, exhibited enhanced biofilm formation on plastic surfaces. In summary, Gtr1 and Rhb1 link nutrient sensing and biofilm formation and this connectivity may have evolved to enhance the competitiveness of *C. albicans* in niches where there is intense competition with other microbes for space and nutrients.

**IMPORTANCE** *Candida albicans* is the major fungal pathogen of humans and is responsible for a wide range of infections, including life-threatening systemic infections in susceptible hosts. Target of rapamycin complex 1 (TORC1) is an essential regulator of metabolism in this fungus, and components of this complex are under increased investigation as targets for new antifungal drugs. The present study characterized the role of *GTR1*, encoding a putative GTPase, in TORC1 activation. This study shows that *GTR1* encodes a protein required for activation of TORC1 activity in response to amino acids and regulation of nitrogen starvation responses. *GTR1* mutants show increased cell-cell adhesion and biofilm formation and increased expression of genes involved in these processes. This study demonstrates that starvation responses and biofilm formation are coregulated by *GTR1* and suggests that these responses are linked to compete with the microbiome for space and nutrients.

## Abstract (precis)

*Candida albicans* is the major fungal pathogen of humans and is responsible for a wide range of infections, including life-threatening systemic infections in susceptible hosts. Target of rapamycin complex 1 (TORC1) is an essential regulator of metabolism in this fungus, and components of this complex are under increased investigation as targets for new antifungal drugs. The present study characterized the role of *GTR1*, encoding a putative GTPase, in TORC1 activation. This study shows that *GTR1* encodes a protein required for activation of TORC1 activity in response to amino acids and regulation of nitrogen starvation responses. *GTR1* mutants show increased cell-cell adhesion and biofilm formation and increased expression of genes involved in these processes. This study demonstrates that starvation responses and biofilm formation are coregulated by *GTR1* and suggests that these responses are linked to compete with the microbiome for space and nutrients.

## INTRODUCTION

Of the clinically relevant *Candida* species, *Candida albicans* is responsible for the vast majority of human infections (1). *C. albicans* is a polymorphic fungus that can change among yeast, pseudohyphal, and true hyphal forms (2). This polymorphic nature contributes to its ability to colonize diverse niches and form biofilms and is a critical factor in the virulence of *C. albicans* (3). The yeast-to-hyphal switch is induced by a number of environmental cues, including a shift to 37°C and alkaline pH that triggers the G-protein Ras1, which initiates a cAMP signaling cascade to activate the transcription factor Efg1 (3, 4). Recently, Tor1 (target of rapamycin) kinase has been implicated in the regulation of morphology in *C. albicans* (5–7). Tor1 is an essential component of target of rapamycin complex 1 (TORC1) and is a central regulator in a nutrient-sensing pathway conserved in eukaryotic cells. It was first identified in *Saccharomyces cerevisiae* in 1991 following an analysis of mutations conferring resistance to the drug rapamycin (8). In *C. albicans*, TORC1 has been implicated in negative regulation of filamentous growth (5, 7, 9, 10). Inhibition of TORC1 results in activation of the GATA transcription factor Brg1, which is involved in modification of the promoters of hypha-specific genes and blocking recruitment of the Nrg1-Tup1 transcriptional repressor complex (5, 7). TORC1 has also been implicated in the regulation of adhesion gene expression and biofilm formation (11, 12).

Activation of TORC1 requires the activity of several small G proteins. The first of these identified was Rheb1, a positive regulator of mammalian Tor (mTor) which is activated by binding of GTP, and the resulting Rheb/GTP complex stimulates the kinase activity of mTor (13). In *C. albicans*, deletion of the orthologous *RHB1* gene enhances sensitivity to rapamycin, suggesting association with the TORC1 signaling pathway (14). Furthermore, Chowdhury et al. have shown that Rhb1 is required for the phosphorylation of ribosomal protein S6, a downstream target of the TORC1 pathway (15). It has been shown that Rhb1 is involved in nitrogen starvation-induced morphogenesis, possibly by controlling the expression of Mep2, a permease and ammonium sensor (16, 17).

In *S. cerevisiae*, the G-protein complex Gtr1/Gtr2 activates Tor1 in response to amino acid signals via the guanine nucleotide exchange factor Vam6 (18, 19). Gtr1 is a member of the RagA subfamily of the GTPase superfamily and is a GTP-binding protein that is essential for amino acid signaling and TORC1 activation in yeast (20). Characterization of mutants unable to escape from rapamycin-induced growth arrest resulted in the identification of the exit-from-G0 complex as an important activator of TORC1. This complex, consisting of Ego1, Ego3, Gtr1, and Gtr2, is vacuole bound and thought to signal amino acid levels to TORC1 via the Gtr1 guanine nucleotide exchange factor Vam6 (18). Homologs of these exist in *C. albicans* but have not yet been characterized. Recently, we have shown that mutants defective in Gtr1, but not in another TORC1-activating GTPase, Rhb1, are defective in the activation of TORC1 in response to phosphate (21). The present study further characterized the role of *GTR1* in the activation of TORC1, characterized the phenotypes regulated by this GTPase, and compared this activity with that of Rhb1. We showed that these GTPases play an important role in linking nutrient starvation responses with signals to enhance colonization, which may have important implications for the lifestyle of this commensal yeast.

## RESULTS

### GTR1 is required for phosphorylation of ribosomal protein S6 and rapamycin resistance.

**FIG 1:**
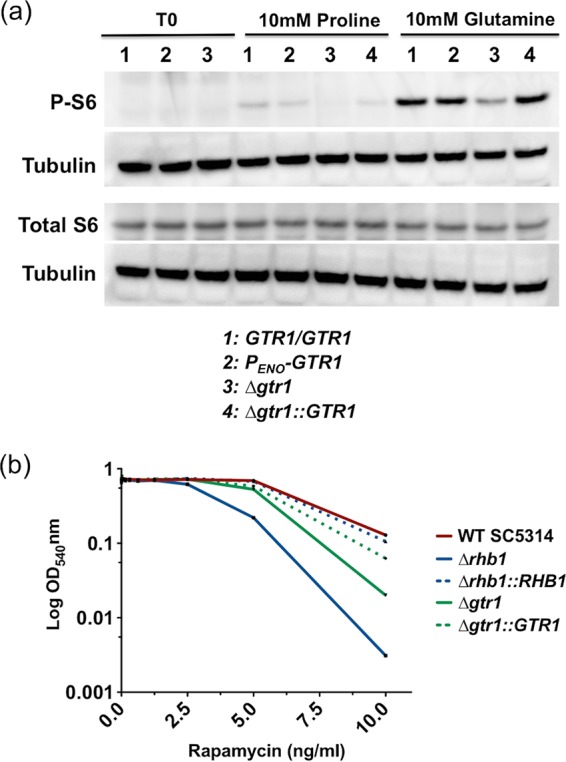
(a) Analysis of the phosphorylation status of ribosomal protein S6 in response to nitrogen stimulation. Following 4 h of nutrient deprivation (YNB without amino acids and ammonium sulfate, 1% glucose), cells were treated with 10 mM glutamine or 10 mM proline and assayed for the presence of total S6 and P-S6. Tubulin was used as a loading control. Lanes: 1, WT *C. albicans* SC5314; 2, P_*ENO*_-*GTR1* derivative; 3, homozygous *gtr1/gtr1* mutant; 4, heterozygous *GTR1/gtr1* mutant. (b) Analysis of rapamycin susceptibility by broth dilution. Rapamycin was diluted from 10 to 0.02 ng/ml. Strains were grown overnight in wells, and growth was assessed by measuring OD_540_. Similar results were obtained in replicate assays.

To determine if *GTR1* is required for TORC1 activation in response to nitrogen stimulation, we carried out an assay to determine the phosphorylation state of ribosomal protein S6 (RPS6), a component of the 40S ribosomal subunit, which has previously been shown to be phosphorylated by TORC1 in response to Rhb1 signaling. Stimulation of wild-type (WT) SC5314 or a derivative strain where *GTR1* was overexpressed from the constitutive *ENO1* promoter (P_*ENO1*_) with 10 mM proline or 10 mM glutamine allowed detection of phosphorylated RPS6 (Fig. 1a). This signal was reduced in a homozygous *gtr1/gtr1* mutant derivative, indicating that Gtr1 has a role in the transduction of nitrogen availability signals via TORC1. Mutants defective in the TOR pathway also exhibit increased susceptibility to rapamycin. The *gtr1/gtr1* mutant exhibited increased susceptibility to rapamycin across a range of concentrations (5 to 10 ng/ml; Fig. 1b). However, the degree of increased susceptibility was greater in a *rhb1/rhb1* mutant.

### GTR1 and RHB1 regulate nitrogen starvation responses.

**FIG 2:**
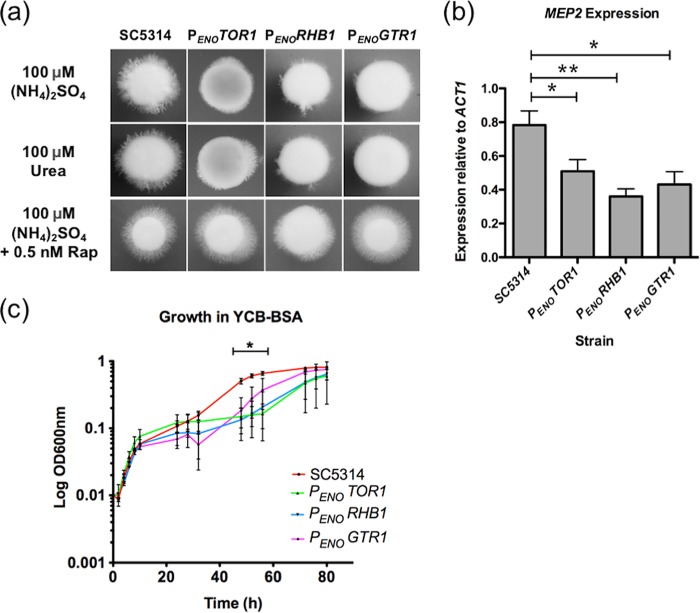
Overexpression of *TOR1*, *GTR1*, and *RHB1* with the *ENO1* promoter. (a) Suppression of hyphal growth on YNB supplemented with 100 µM urea or 100 µM (NH_4_)_2_SO_4_. Rapamycin (0.5 nM) could reverse this phenotype. (b) *MEP2* expression in the strains indicated was measured by qRT-PCR and expressed relative to *ACT1* expression. Stars indicate significant differences from the WT (ANOVA; *, *P* < 0.05; **, *P* < 0.01) (c) Growth in YCB-BSA was measured (OD_600_) over an 80-h period. The star indicates time points where significant differences in biomass were detected (ANOVA, *P* < 0.01).

TORC1 activation by nutrients suppresses nitrogen starvation responses in *C. albicans*, and we compared the abilities of Rhb1 and Gtr1 to suppress these responses. We first compared the nitrogen starvation-induced filamentous growth of WT SC5314 with that of derivatives with *GTR1*, *RHB1*, or *TOR1* itself overexpressed from the enolase promoter (P_*ENO*_). On solid yeast extract-peptone-dextrose (YEPD) medium, these strains were indistinguishable from WT SC5314. On medium containing 100 µM urea or 100 µM (NH_4_)_2_SO_4_ as the sole nitrogen source, WT SC5314 exhibits a hyphal fringe surrounding the entire colony. This fringe was partially suppressed in the P*_ENO_TOR1*, P*_ENO_RHB1*, and P*_ENO_GTR1* strains. Inhibition of TORC1 activity with a subinhibitory concentration of rapamycin (0.5 nM) resulted in reversal of the inhibition of hyphal growth in all P_*ENO*_ strains, resulting in a phenotype similar to that of WT SC5314. Deletion of *GTR1* or *RHB1* did not significantly affect this nitrogen starvation-induced filamentation (data not shown). Filamentous growth in response to nitrogen limitation is known to involve the transceptor Mep2. Tsao et al. (14) showed that the expression of *MEP2* is suppressed in a strain overexpressing *RHB1*. In the present study, the expression of *MEP2* mRNA was found to be significantly lower in all P_*ENO1*_ strains (analysis of variance [ANOVA], *P* < 0.05, Fig. 2b). Increased TORC1 activity has also been shown to suppress growth in the presence of alternative nitrogen sources such as bovine serum albumin (BSA) (16). Following growth in yeast carbon base medium supplemented with BSA (YCB-BSA), P*_ENO_TOR1*, P*_ENO_RHB1*, and P*_ENO_GTR1* exhibited significantly less biomass (optical density at 600 nm [OD_600_]) from 48 h than WT SC5314 (ANOVA, *P* < 0.01; Fig. 2c).

### Formation of hyphae and flocculation.

**FIG 3:**
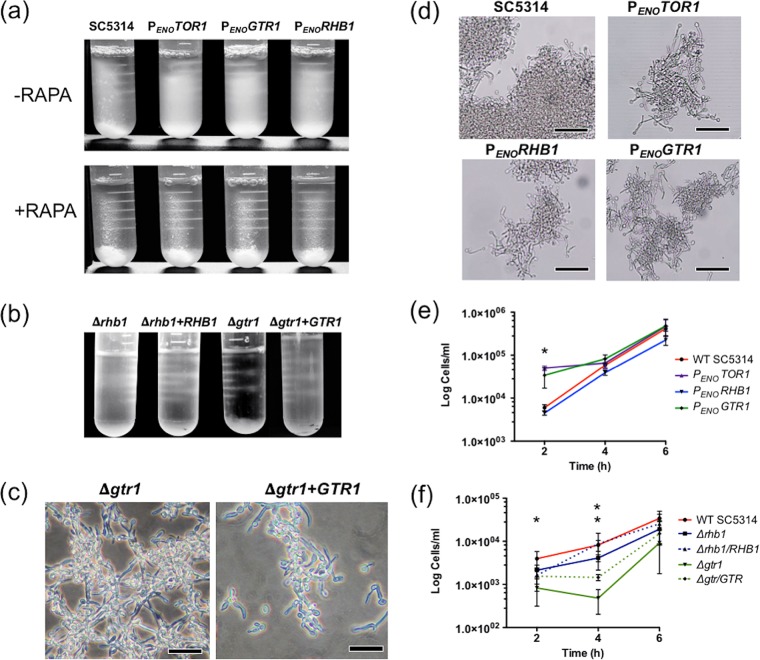
Analysis of flocculation and filamentous growth. (a) Overexpression of *GTR1*, *RHB1*, and *TOR1* with the *ENO1* promoter (P_*ENO*_) results in reduced aggregation and flocculation in Spider medium. This could be reversed by the addition of rapamycin (0.5 nM). (b) The *gtr1/gtr1* mutant (Δ*gtr*) exhibits more rapid flocculation in Spider medium following static incubation than the *rhb1/rhb1* mutant (Δ*rhb1*). (c) Microscopic analysis of the flocculate from panel b showing that the Δ*gtr* mutant was extensively pseudohyphal. Bars, 20 µm. (d) *C. albicans* strains that overexpress *GTR1*, *RHB1*, or *TOR1* produced true hyphae in YEPD supplemented with 10% (vol/vol) serum but flocculated less than WT SC5314. Bars, 40 µm. (e) Quantitative analysis of the numbers of nonflocculating free yeast cells in cultures of the strains indicated in YEPD supplemented with 10% (vol/vol) serum. The star indicates a significant difference between the WT strain and the P_*ENO*_-*GTR1* and P_*ENO*_-*TOR1* strains (*t* test, *P* < 0.05). (f) Quantitative analysis of the numbers of nonflocculating free yeast cells in cultures of the *gtr1/gtr1* mutant (Δ*gtr*), the *rhb1/rhb1* mutant (Δ*rhb1*), and the complemented derivatives in YEPD supplemented with 10% (vol/vol) serum. The single star indicates a significant difference between the WT and the Δ*gtr* mutant at 2 h, and the double stars indicate a difference between the Δ*gtr* and Δ*rhb1* mutants at 4 h (*t* test, *P* ≤ 0.05).

Bastidas et al. (11) demonstrated that *C. albicans* TORC1 regulates the expression of adhesins and that this can facilitate the flocculation of *C. albicans* cells in liquid Spider medium. We first compared the flocculation of the P_*ENO*_ strains described above to that of WT SC5314 in Spider medium. Strains were incubated with shaking at 200 rpm, after which they were left stationary for 20 min to allow cells to flocculate (Fig. 3a). WT SC5314 flocculated and settled at the bottom of the tube more rapidly (within 20 min) than the P_*ENO*_ strains. Inhibition of TORC1 with 0.5 nM rapamycin resulted in the flocculation of all of the strains (Fig. 3a). Next, we carried out the same assay with SC5314 and the *gtr1/gtr1* and *rhb1/rhb1* mutants. Unexpectedly, the *gtr1/gtr1* mutant exhibited rapid flocculation upon removal form the shaking incubator (within 10 min; Fig. 3b), whereas the complemented *gtr1/GTR1* culture remained turbid at this time. Visual inspection of these clumps revealed a greater abundance of pseudohyphae in the flocculate from *gtr1/gtr1* mutant cultures (Fig. 3c). The *rhb1/rhb1* mutant did not exhibit this phenotype (data not shown).

In YEPD supplemented with 10% (vol/vol) serum at 37°C, all of the strains (including the *gtr1/gtr1* and *rhb1/rhb1* mutants and the P_*ENO*_ fusion strains) could form true hyphae. However, following microscopic observation, it was noted that the hyphae formed by the P_*ENO*_ strains formed smaller, loosely adherent clumps compared to the masses of hyphae formed by WT SC5314 (Fig. 3d). Additionally, it was observed that the number of free, nonadherent cells in hyphal cultures of the P_*ENO*_ strains was greater than that in WT SC5314. To quantify the number of free, nonadherent cells in these cultures, we developed a quantitative filtration assay to enumerate free cells in the culture (Materials and Methods). Following a 2-h incubation in 10% serum, filtrates of P*_ENO_TOR1* and P*_ENO_GTR1* cultures had significantly higher counts (*P* < 0.05; 3.6-fold and 3.2-fold change, respectively) of free yeast cells than did those of WT SC5314 (Fig. 3e). Addition of 0.5 nM rapamycin to the cultures could reverse this phenotype, with all strains exhibiting high levels of aggregation and reduced free-cell counts of ~10^3^/ml (data not shown).

Both the *gtr1/gtr1* and *rhb1/rhb1* mutants were capable of forming WT levels of true hyphae in YPD supplemented with 10% serum. Visual inspection revealed that the *gtr1/gtr1* and *rhb1/rhb1* mutants formed greater massed clumps of hyphae than WT SC5315 (Fig. S1). Enumeration of free yeast cells in these cultures revealed that the *gtr1/gtr1* and *rhb1/rhb1* mutant cultures contained fewer free yeast cells than WT SC5314 (Fig. 3f). These differences were greatest in the *gtr1/gtr1* mutant and were significant in both mutants at 4 h (*gtr1/gtr1*, *P* = 0.038; *rhb1/rhb1*, *P* = 0.05).

10.1128/mSphere.00477-17.5 FIG S1  Microscopic analysis of hyphal flocculates in cultures of WT SC5314, the *gtr1* and *rhb1* homozygous mutants, and complemented derivatives. Download FIG S1, PDF file, 2.1 MB. Copyright © 2017 Flanagan et al. 2017 Flanagan et al. This content is distributed under the terms of the Creative Commons Attribution 4.0 International license.

10.1128/mSphere.00477-17.6 FIG S2  Interaction map showing enriched categories of genes in the *gtr1/gtr1* mutant that are upregulated (red) or downregulated (blue) relative to WT SC5314, generated in Cytoscape 3.2.1. The GSEA PreRanked tool was used to investigate whether our data sets were enriched for particular genes present in published data sets. The map shows enrichments that were highly significant (*P* value of <0.05 and FDR *Q* value cutoff set at 0.05). Nodes with significant overlaps in gene content are connected by lines by using the overlap coefficient in Cytoscape. Download FIG S2, PDF file, 1.5 MB. Copyright © 2017 Flanagan et al. 2017 Flanagan et al. This content is distributed under the terms of the Creative Commons Attribution 4.0 International license.

### Transcript profiling of *gtr1*/*gtr1*.

**FIG 4:**
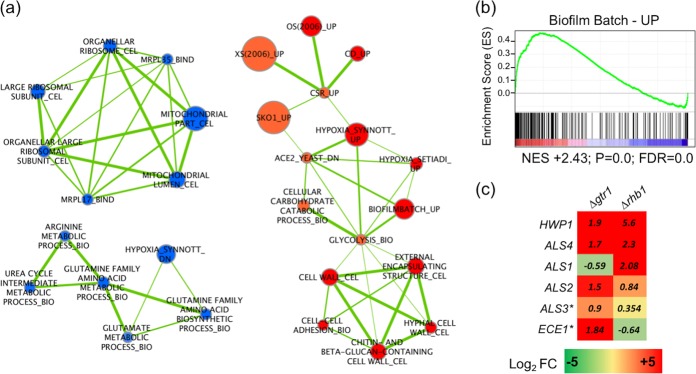
Transcript profiling of the *gtr1/gtr1* mutant in liquid YEPD at 37°C. (a) Interaction network generated in Cytoscape from highly significantly enriched categories of genes (*P* < 0.05 and FDR *Q* value of <0.05) identified by GSEA. Each node in the map represents a cluster of genes associated with a specific function, with red indicating genes upregulated and blue indicating genes downregulated. Categories in orange were upregulated in the *gtr1/gtr1* mutant but downregulated in the *rhb1/rhb1* mutant. OS, osmotic stress; XS, oxidative stress; CSR, core stress response; CD, cell density. Only representative features are shown (the full map is presented in Fig. S2). (b) Genes corresponding to those induced during biofilm batch growth (Biofilm Batch - UP) by Sellam et al. (42) were significantly upregulated. A positive normalized enrichment score (NES) indicated enrichment in upregulated genes; a *P* value and FDR of 0.0 represents <0.001. (c) Regulation of cell surface and secreted genes in the *gtr1/gtr1* and *rhb1/rhb1* mutants. Genes marked with asterisks were shown previously to be induced in the *rhb1/rhb1* mutant during growth in SD medium (16). FC, fold change.

Transcript profiling of the *gtr1/gtr1* mutant during batch growth in YPD was performed, and gene set enrichment analysis (GSEA) was used to investigate the main functional categories of genes regulated (Fig. 4a). Significant upregulation of genes expressed during biofilm growth was observed (Fig. 4b), including cell surface and secreted proteins with roles in adhesion (*HWP1*, *ALS2*, *ALS3*, *ALS4*, and *ECE1*; Fig. 4c). Genes involved in carbohydrate catabolism were also induced, in addition to those involved in several stress responses (hypoxia, oxidative stress), including those regulated by the transcription factor Sko1 (22–24). Amino acid biosynthesis and ribosome biogenesis were also downregulated (Fig. 4a). Analysis of the *rhb1/rhb1* mutant data set revealed a similar set of significantly enriched gene categories, including reduced ribosome biogenesis, amino acid biosynthesis, and increased expression of biofilm- and cell wall-associated genes (Fig. 4c; see Fig. S3 in the supplemental material). Although we did not detect increased expression of *ECE1* and *ALS3* in the *rhb1/rhb1* mutant, Chen et al. (16) reported that these genes were induced in this mutant during growth in SD medium (16). Comparative analysis of the enrichment patterns in the *gtr1/gtr1* and *rhb1/rhb1* mutants revealed differential expression of several gene categories, including oxidative stress responses, carbohydrate catabolism, and genes regulated by the Sko1 transcriptional regulator (orange in Fig. 4a; Fig. S4). Phenotypic analysis revealed that resistance to oxidative stress (H_2_O_2_) was similar to that of the WT in both mutants (Fig. S4). As Sko1 is a regulator of the cell wall damage response, we compared the susceptibility of the mutants to the cell wall-damaging agents calcofluor white and Congo red. The *rhb1/rhb1* mutant exhibited increased susceptibility to both agents, whereas the *gtr1/gtr1* mutant exhibited WT levels of susceptibility (Fig. S4).

10.1128/mSphere.00477-17.7 FIG S3  Interaction map showing enriched categories of genes in the *rhb1/rhb1* mutant that are upregulated (red) or downregulated (blue) relative to WT SC5314, generated in Cytoscape 3.2.1. The GSEA PreRanked tool was used to investigate whether our data sets were enriched for particular genes present in published data sets. The map shows enrichments that were highly significant (*P* value of <0.05 and FDR *Q* value cutoff set at 0.05). Nodes with significant overlaps in gene content are connected by lines by using the overlap coefficient in Cytoscape. Download FIG S3, PDF file, 1.8 MB. Copyright © 2017 Flanagan et al. 2017 Flanagan et al. This content is distributed under the terms of the Creative Commons Attribution 4.0 International license.

10.1128/mSphere.00477-17.8 FIG S4  (a) Comparison of the enrichment patterns of genes in the categories SKO1_Up and XS(2006)_Up in the *gtr1/gtr1* and *rhb1/rhb1* mutants identified by GSEA. (b) The *rhb1* mutant shows increased susceptibility to the cell wall-damaging agents Congo red and calcofluor white. No significant change in susceptibility to H_2_O_2_ was detected in any of the strains analyzed. Download FIG S4, PDF file, 0.7 MB. Copyright © 2017 Flanagan et al. 2017 Flanagan et al. This content is distributed under the terms of the Creative Commons Attribution 4.0 International license.

### Biofilm formation analysis.

**FIG 5:**
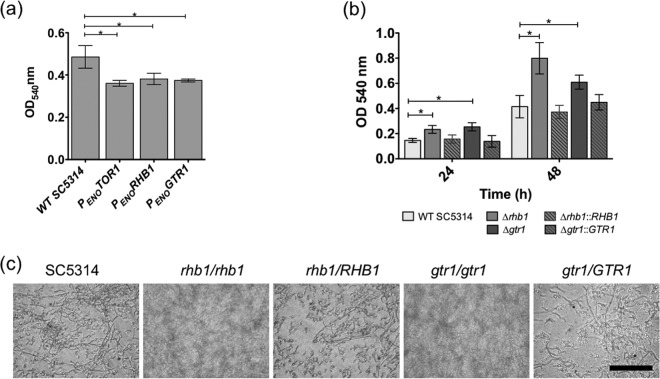
Biofilm formation in *C. albicans* and mutant derivatives. (a) Biofilm mass was compared in SC5315 and the P*_ENO_TOR1*, P*_ENO_RHB1*, and P*_ENO_GTR1* derivatives with the crystal violet assay following 48 h of growth in Spider medium at 37°C. The P_*ENO*_ strains formed significantly less biofilm. A star indicates a *P* value of <0.05 (ANOVA with Dunnett’s multiple-comparison test). (b) Biofilm mass was compared in SC5315 and the *gtr1/gtr1* mutant (Δ*gtr*), the *rhb1/rhb1* mutant (Δ*rhb1*), and complemented derivatives with the crystal violet assay following 24 and 48 h of growth in Spider medium at 37°C. The Δ*rhb1* and Δ*gtr* mutant strains formed significantly more biofilm. A star indicates a *P* value of <0.05 (ANOVA with Dunnett’s multiple-comparison test). (c) Light micrographs of 48-h biofilms of SC5314, the *gtr1/gtr1* mutant, the *rhb1/rhb1* mutant, and complemented derivatives. Bar, 50 µm.

Because of the effects of these mutations on aggregation, flocculation, and the expression of biofilm-related genes, we analyzed biofilm formation in 96-well plates by using a crystal violet assay. Following a 48-h incubation, the P*_ENO_TOR1*, P*_ENO_RHB1*, and P*_ENO_GTR1* strains showed significantly less biofilm formation than WT SC5314 (ANOVA, *P* = 0.02; Fig. 5a). Next, we analyzed biofilm formation in the *gtr1/gtr1* and *rhb1/rhb1* mutants. Following 24 and 48 h of incubation, the *gtr1/gtr1* and *rhb1/rhb1* mutants formed more biofilm than WT SC5314 (ANOVA, *P* < 0.01) and the respective complemented derivatives (Fig. 5b). At 48 h, the *rhb1/rhb1* mutant exhibited the largest increase in the level of biofilm formation compared with WT SC5314 and the complemented *RHB1* strain. By light microscopic analysis, it was observed that the *gtr1/gtr1* and *rhb1/rhb1* mutants produced a denser biofilm mass than WT SC5314 and the complemented derivatives (Fig. 5c).

### Analysis of virulence in the *Galleria mellonella* model.

**FIG 6:**
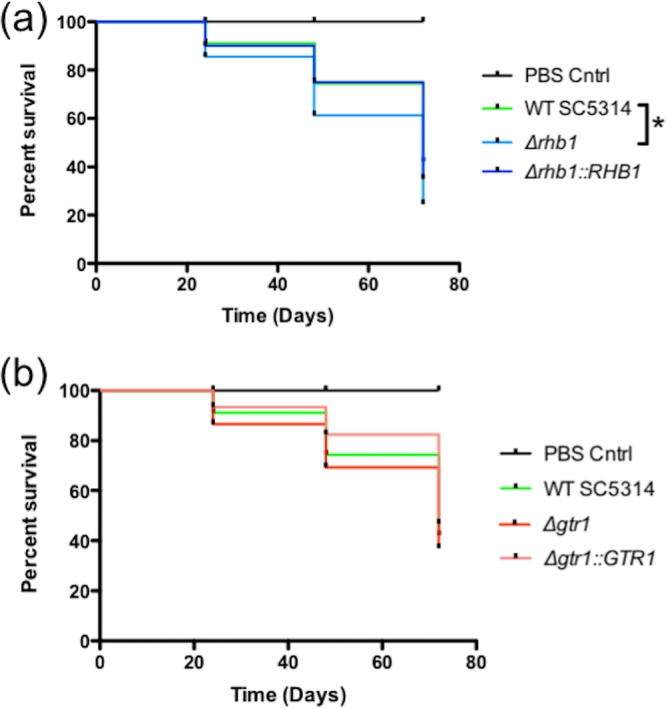
The virulence of SC5314, the *gtr1/gtr1* mutant, the *rhb1/rhb1* mutant, and their complemented derivatives was analyzed in the *G. mellonella* model of infection. Survival curves were generated with the Prism software package. A star indicates a significant difference between survival curves (log rank Mantel-Cox test, *P* = 0.032).

Virulence in the *G. mellonella* model was examined in SC5314, the *gtr1/gtr1* and *rhb1/rhb1* homozygous mutants, and their complemented derivatives (Fig. 6). The *rhb1/rhb1* mutant exhibited marginally greater virulence than SC5314, and this difference was significant (log rank Mantel-Cox test, *P* = 0.032). WT virulence was restored in the complemented *rhb1/RHB1* mutant. The virulence of the *gtr1/gtr1* mutant was also marginally greater than that of the complemented derivative but not significantly different from that of WT SC5314.

## DISCUSSION

Tor1 is an essential component of the TORC1 complex and plays a central role in the regulation of metabolism and nutrient acquisition in eukaryotic organisms. TORC1 controls cellular activity on the basis of the availability of nutrients, the presence of which is relayed to the complex by several small G proteins, including Gtr1 and Rhb1. For *C. albicans*, a mucosal commensal and opportunistic pathogen, responding to nutrient availability and adapting cellular activity to those signals is likely crucial for successful colonization of the host. During most stages of the *C. albicans* life cycle, which largely consists of vegetative growth on mucosal surfaces, effectively competing with the bacterial microbiome for nutrients is crucial in the struggle for survival. In this study, we characterized the GTPase Gtr1 and compared its role with that of another GTPase, Rhb1, in the control of TORC1-regulated phenotypes and gene expression patterns. This is the first study to confirm the role of *C. albicans* Gtr1 in the activation of TORC1 in response to nitrogen availability. Previous studies have implicated Gtr1 in phosphate sensing, and the present study demonstrates that Gtr1 is required for activation of TORC1 in response to proline and glutamine. Chowdhury et al. (15) showed that Rhb1 is required for TORC1-mediated phosphorylation of RPS6 in nitrogen-rich medium, suggesting some overlap in the roles of Rhb1 and Gtr1 in nitrogen sensing. However, the localization of these proteins is likely different and they may play a role in the detection of the presence of nitrogen sources at the plasma membrane and vacuole, respectively (25, 26). We also noticed some differences in the transcriptional responses of both mutants, specifically, the oxidative-stress-regulated gene set and expression of the Sko1 regulon (24, 27). *SKO1* mutants are rapamycin resistant, indicating that Sko1 interacts with the TORC1 pathway (28). The effects of the *Δgtr1* and Δ*rhb1* mutations on the Sko1-regulated gene set support this hypothesis. However, the *Δgtr1* and *Δrhb1* mutations appear to have opposing effects on Sko1 transcription, indicating that Gtr1 and Rhb1 may differ in some aspects of TORC1 regulation.

The present study shows that both proteins play a role in the regulation of nitrogen starvation responses in response to low ammonium and BSA (Fig. 2), and gene expression patterns in the homozygous mutants also indicate that defective TORC1 signaling also reduces the level of expression of genes involved in ribosome biogenesis and amino acid biosynthesis, which resembles the response of *C. albicans* to rapamycin exposure (11). Rapamycin treatment of *C. albicans* has also been linked to the expression of adhesins and increased flocculation and biofilm formation. TORC1 likely exerts this effect via activation of the transcription factor Sfp1, a repressor of genes required for biofilm formation (12). The present study demonstrates that these phenotypes can be regulated upstream of TORC1 by the GTPases Rhb1 and Gtr1. Modulation of the levels of either gene resulted in hyperflocculation (*Δgtr1*, *Δrhb1*) or hypoflocculation (*P*_*ENO*_ fusions), suggesting that the adhesive characteristics of *C. albicans* are tightly linked to the activity of TORC1. A similar pattern of biofilm hyper- and hypoproduction was also observed with the deletion mutants and *P*_*ENO*_ fusion strains, respectively. Gene expression patterns in both *Δgtr1* and *Δrhb1* mutants showed a significant enrichment for genes expressed during biofilm formation, including the *HWP1* and *ALS* genes, which encode important virulence factors (29, 30). It is interesting to note the connectivity between starvation responses (reduced biosynthesis, increased catabolism) and the activation of adhesive and biofilm phenotypes. It is our hypothesis that the interconnectivity of these responses has evolved in *C. albicans* to enhance the colonization of mucosal surfaces, where competition for both space and nutrients with the bacterial microbiota is an important evolutionary pressure. The nature of this response suggests that when *C. albicans* senses reduced nutrient availability, it may be a signal indicating overgrowth of the surrounding microbiome. The best way to deal with this competition for nutrients may be to maximize the ability to colonize available surfaces. This is perhaps not surprising, as changes in the morphological form of a fungus are often associated with foraging activity, for example, in *S. cerevisiae*, which mounts its pseudohyphal growth program in response to nitrogen starvation (31). In *C. albicans*, which may be under considerable pressure for space and nutrients in the gastrointestinal tract, a biofilm response to reduced TORC1 signaling may enhance its abilities to expand its territory and compete for nutrients. In the case of *RHB1*, this hypothesis is supported by the data from the *G. mellonella* model, which suggest that the mutant strain can more aggressively colonize the hemocoel of the larva.

In summary, both Rhb1 and Gtr1 play complementary roles in the regulation of TORC1 activity, which integrates these signals to regulate not only metabolism but also the ability to colonize surfaces. It is likely that this regulatory program evolved to enhance survival in niches where both space and nutrients are in great demand.

## MATERIALS AND METHODS

### *Candida* strains and culture conditions.

The strains used in the present study are described in Table S1. Strains were routinely cultured on YEPD agar (1% [wt/vol] yeast extract, 2% [wt/vol] peptone, 2% [wt/vol] glucose) and incubated overnight at 37°C. For long-term storage, stocks were maintained at −80°C on Microbank beads (Pro-Lab Diagnostics, ON, Canada). For broth cultures, a single colony was subcultured from these plates into YEPD broth overnight at 37°C with shaking at 200 rpm. Medium consisting of yeast nitrogen base (YNB) without amino acids and ammonium sulfate (0.17%) was used for various assays and was supplemented with nitrogen sources and glucose as indicated.

10.1128/mSphere.00477-17.1 TABLE S1  Details of the *C. albicans* strains used in this study. Download TABLE S1, DOCX file, 0.02 MB. Copyright © 2017 Flanagan et al. 2017 Flanagan et al. This content is distributed under the terms of the Creative Commons Attribution 4.0 International license.

### Ribosomal protein S6 phosphorylation assay.

To assay the phosphorylation status of ribosomal protein S6A (RPS6), cells were grown in a nutrient-poor environment (YNB [without amino acids and ammonium sulfate] and 1% [wt/vol] glucose) for 4 h to induce starvation. Following incubation, water, 10 mM glutamine, or 10 mM proline was added to the medium. The assay was carried out as described by Chowdhury et al. (15). Membranes were probed for phosphorylated S6 (P-S6) by using anti-phospho-(Ser/Thr) Akt substrate rabbit polyclonal antibody (Cell Signaling Technology, Inc., catalog no. 9611). Total S6 was monitored with an anti-S6 sheep polyclonal antibody (R&D Systems catalog no. AF5436). Anti-tubulin rat monoclonal antibody (Abcam, Inc., catalog no. ab6161) was used to monitor the loading controls.

### *Candida* genetics.

Transformations of *C. albicans* and selection for nourseothricin resistance were carried out as described by Moran et al. (32). To overexpress selected genes, the *C. albicans* enolase promoter (P_*ENO*_) was placed upstream with plasmid pNAT-ENO1 as described by Milne et al. (33). With primers to target the specific genes (Table S2), P_*ENO*_ was amplified and integrated upstream of *TOR1*, *RHB1*, and *GTR1* following transformation with the relevant construct. Integration was confirmed with a *P*_*ENO*_-specific primer (ENOp Con F1) and a gene-specific primer (Table S1). Elevated levels of gene expression (at least 10-fold) were confirmed by quantitative reverse transcription (qRT)-PCR in each case (data not shown).

10.1128/mSphere.00477-17.2 TABLE S2  Sequences of the oligonucleotides used in this study Download TABLE S2, DOCX file, 0.02 MB. Copyright © 2017 Flanagan et al. 2017 Flanagan et al. This content is distributed under the terms of the Creative Commons Attribution 4.0 International license.

*GTR1* (C2_08600W) knockout constructs were generated by a PCR-based method with primers in Table S1 (34). Primers GTR1_F1 and GTR1_R1 were used to amplify the *SAT1* cassette, which was used in transformations as previously described (35). Confirmation of the homozygous deletion was carried out with a combination of PCR primers complementary to regions flanking the deletion (GTR1_Flank1 and -2) and within the deletion (GTR1_IntF and -R). To complement the mutant, *GTR1* was amplified with primers GTR1_Reint F1 and R1 (Table S2) and cloned by using XhoI and HindIII restriction endonuclease cleavage and ligation to pCDRI, and this plasmid was transformed into the *gtr1/gtr1* homozygous mutant as previously described (36).

### Hyphal growth, aggregation, and flocculation assays.

Single colonies were grown in YEPD broth overnight at 37°C with shaking at 200 rpm. Following incubation, cells were washed with phosphate-buffered saline (PBS) and resuspended in 1 ml of PBS. A 1-ml suspension containing 2 × 10^6^ cells/ml was prepared, of which 2 µl was spotted onto SLD agar [0.17% YNB without amino acids, 0.5% NH_4_(SO_4_)_2_, 0.1% glucose, 1.4% (wt/vol) agar] plates containing various nitrogen and amino acid sources and incubated for 7 days at 37°C. For liquid assays, cells were grown overnight in YEPD at 30°C with shaking at 200 rpm, washed in PBS, counted, and resuspended in 25 ml of YEPD supplemented with 10% (vol/vol) fetal bovine serum (Sigma) at a density of 2 × 10^6^/ml. To quantify the number of free cells in the culture, a 1-ml sample of culture was filtered through a 10-µm Versapor membrane. Filtrates were centrifuged at 20,000 × *g* for 1 min, and the pellet was resuspended in 100 µl. Cells were counted with an improved Neubauer hemocytometer. For visual assessment of flocculation, cells were grown in Spider medium (1% nutrient broth, 0.2% K_2_HPO_4_ 1% mannitol, pH 7.2). Cultures were incubated in Spider medium at 37°C at 200 rpm for 2 h with and without the addition of 0.5 nM rapamycin as indicated. After incubation, samples were removed from the incubator, vortexed briefly, and placed into a test tube rack and the level of flocculation was recorded by photography at the times indicated.

### Biofilm formation assays.

Biofilm mass was quantified with crystal violet (37). Cells were grown in YEPD at 37°C overnight with shaking at 200 rpm. Following overnight incubation, 100 µl was removed and transferred to YNB with 100 mM glucose and incubated overnight at 37°C with shaking at 200 rpm. Cells were then washed in PBS and resuspended in 1 ml of YNB with 100 mM glucose at a density of 2 × 10^6^/ml. A 100-µl volume of each strain was placed in triplicate into the wells of a 96-well plate and incubated at 37°C for 90 min. Following incubation, the medium was aspirated and the cells were washed twice with 150 µl of PBS. Spider medium was placed into each well, and the plates were incubated at 37°C for 24 or 48 h, respectively. Wells were washed three times with 200 µl of sterile PBS to remove nonadherent cells, and 110 µl of 0.4% (vol/vol) crystal violet was added to each well and stained at room temperature for 45 min. The crystal violet was removed, and each well was washed with 200 µl of sterile H_2_O three times. The wells were destained with 200 µl of 95% (vol/vol) ethanol for 45 min. A 100-µl aliquot of each suspension was transferred to a new 96-well plate, and the absorbance at 595 nm was measured with a Tecan Genios plate reader (Tecan, Switzerland). Differences in biofilm formation were analyzed by ANOVA with Dunnett’s multiple-comparison test in Prism version 6 (GraphPad, San Diego, CA).

### Gene expression profiling.

For qRT-PCR, RNA was extracted with the RNeasy minikit (Qiagen) as previously described (36). Extracted RNA was treated with the TURBO DNase kit (Applied Biosystems, Life Technologies, Inc.). cDNA synthesis was performed with oligo(dT) (Promega, Madison, WI) and Superscript III (Invitrogen, Life Technologies, Inc.). qRT-PCR was carried out on the Applied Biosystems 7500 Fast Real Time PCR System with the Fast SYBR green master mix (Applied Biosystems, Life Technologies, Inc.) in accordance with the manufacturer’s instructions. Primers targeting *ACT1* and *MEP2* with optimized amplification efficiencies were used (Table S2). Results of three biological replicates were analyzed in Prism version 6 (GraphPad, San Diego, CA).

For whole-genome transcript profiling, RNA was extracted from cells grown to an OD_600_ of 0.8 in YEPD broth at 37°C. Cell pellets were snap-frozen in liquid N_2_ and disrupted with a Mikro-Dismembrator S (Sartorius Stedim Biotech, Göttingen, Germany), and RNA was purified as described above. WT SC5314 and the *gtr1/gtr1* homozygous mutant were compared with *C. albicans* whole-genome oligonucleotide microarrays (22). A 200-ng aliquot of total RNA was labeled with Cy5 or Cy3 with the two-color low-input Quick Amp labeling kit (Agilent Technologies) in accordance with the manufacturer’s instructions. Array hybridization, washing, scanning, and data extraction were carried out in GenePix Pro 6.1 (Axon) as previously described (36). Four biological replicate experiments were performed, including two dye swap experiments. Raw data were exported to GeneSpring GX13, and signals for each replicate spot were background corrected and normalized by locally weighted scatterplot smoothing transformation. Log_2_ fluorescence ratios were generated for each replicate spot and averaged.

Transcript profiling of the *rhb1/rhb1* homozygous mutant was carried out by RNA sequencing analysis. Stranded RNA libraries were prepared with the TruSeq Stranded Total RNA Library Prep kit (Illumina) and sequenced on the Illumina HiSeq by using a paired-end strategy (2 × 150 bp) yielding a minimum of 15 million reads per sample. Raw sequencing reads were aligned with the *C. albicans* (ASM18296v3) reference transcriptome with kallisto v0.43.1 (38). Sequence reads were aggregated into a count for each gene with *tximport* (39). Differentially expressed genes (false-discovery rate [FDR] of 5%) were identified with DESeq2 (40).

The GSEA PreRanked tool (available at http://www.broadinstitute.org/gsea/index.jsp) was used to investigate whether our data sets were enriched for particular genes present in published data sets. This analysis required the use of a database of publicly available genome-wide data sets (constructed by Andre Nantel, National Research Council of Canada, Montreal), that can be downloaded from the *Candida* Genome Database. To visualize these enrichments and to identify related gene sets, the data were export to Cytoscape 3.2.1. Once the data were exported to Cytoscape, enrichment maps were generated by using the overlap coefficient and selecting only those enrichments that were highly significant (*P* value of <0.05 and FDR *Q* value cutoff set at 0.05). Log_2_ fold changes for each gene relative to the WT are listed in Tables S3 and S4.

10.1128/mSphere.00477-17.3 TABLE S3  Gene expression in strain CCT-D1 (Δ*rhb*) and the *RHB1*-complemented derivative (Δ*rhb RHB1*) versus that in WT SC5314. Values are log_2_ fold changes relative to the WT. Download TABLE S3, XLSX file, 0.7 MB. Copyright © 2017 Flanagan et al. 2017 Flanagan et al. This content is distributed under the terms of the Creative Commons Attribution 4.0 International license.

10.1128/mSphere.00477-17.4 TABLE S4  Gene expression in the *gtr1/gtr1* mutant relative to that in WT SC5314. Values are log_2_ fold changes relative to the WT. Download TABLE S4, XLSX file, 0.7 MB. Copyright © 2017 Flanagan et al. 2017 Flanagan et al. This content is distributed under the terms of the Creative Commons Attribution 4.0 International license.

### *G. mellonella* infection model.

*G. mellonella* infection models were performed as described by Brennan et al. (41). For experimental purposes, larvae weighing 0.2 to 0.3 g were used in each assay. A total of 4× 10^5^ yeast cells in 20 µl of PBS was inoculated into the hemocoel via the left proleg as previously described. Ten larvae were used per treatment, and all groups were placed in a static incubator at 30°C. Data were analyzed with Prism version 6 (GraphPad, San Diego, CA).

### Accession number(s).

All transcript profiling data are available from the NCBI (*gtr1/gtr1*, GEO accession no. GSE104160; *rhb1/rhb1*, BioProject accession no. PRJNA407874).
