# Supplementary figures and images for: The Candida albicans TOR-Activating GTPases Gtr1 and Rhb1 Coregulate Starvation Responses and Biofilm Formation
Source: mSphere. 2017 Nov 15;2(6):e00477-17. doi: 10.1128/mSphere.00477-17 (PMC5687921; doi:10.1128/mSphere.00477-17)

Figure S1.

SC5314

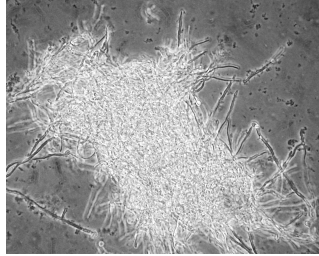

*rhb1/rhb1*

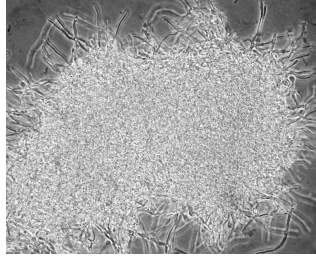

*rhb1/RHB1*

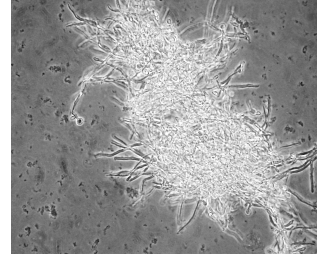

*gtr1/gtr1*

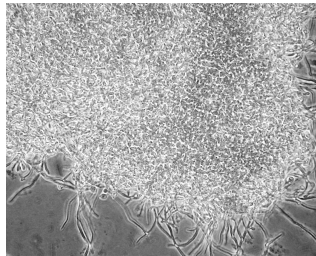

*gtr1/GTR1*

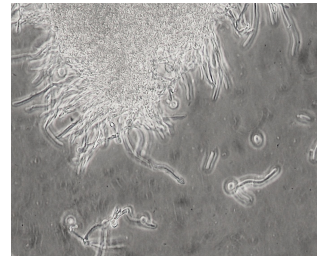

Supplement: FIG S1 [file sph006172401sf5.pdf]

## Figure S3

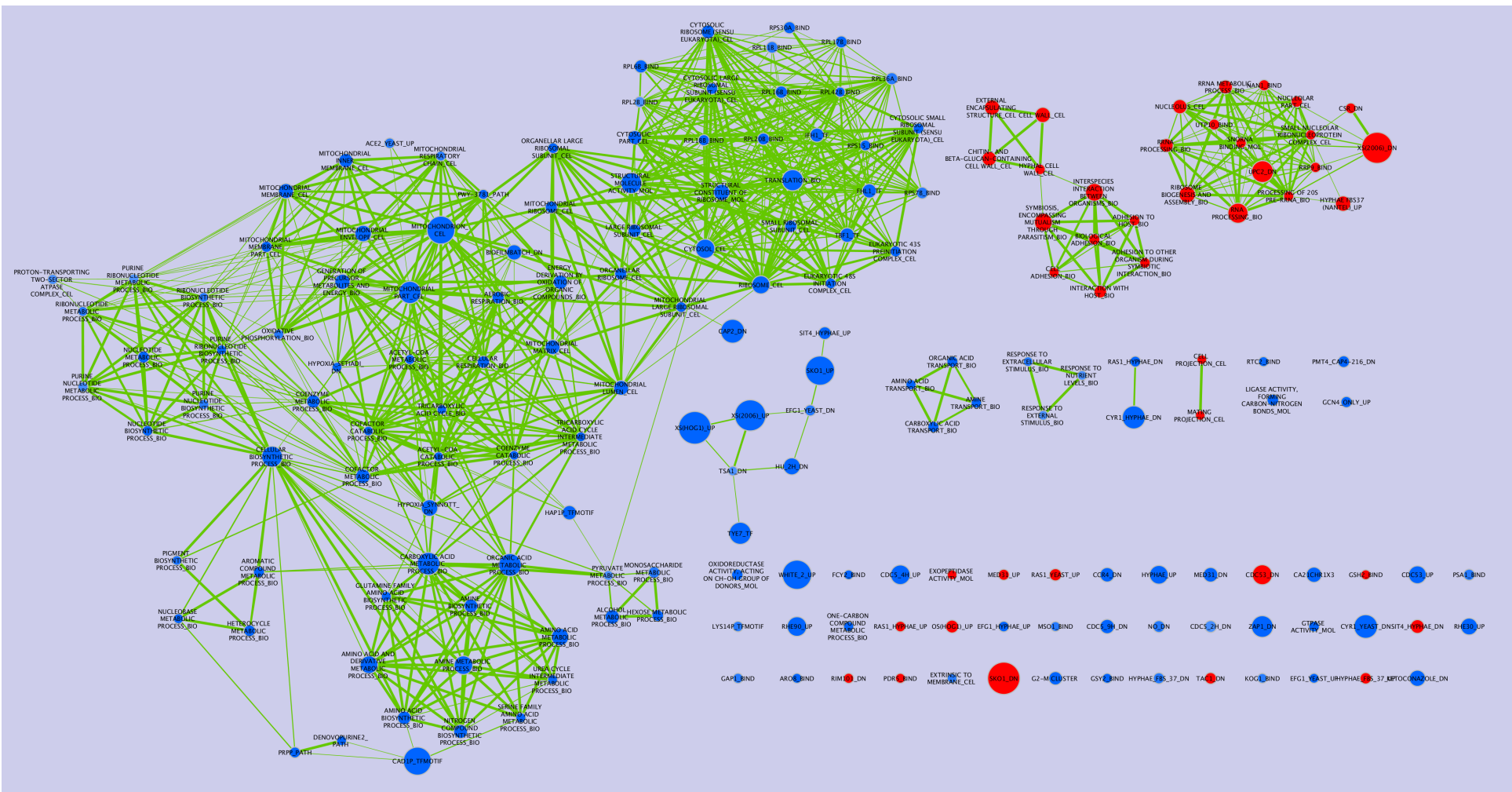

Supplement: FIG S3 [file sph006172401sf7.pdf]

Figure S4

(a)

*gtr1/gtr1*

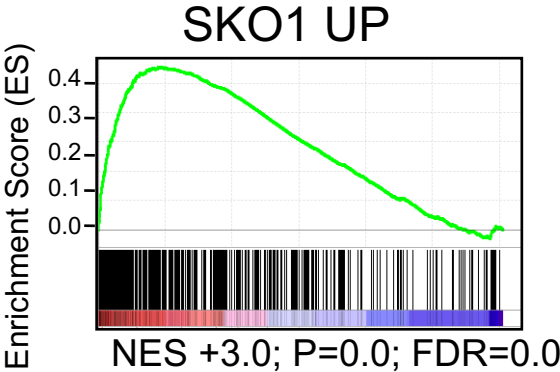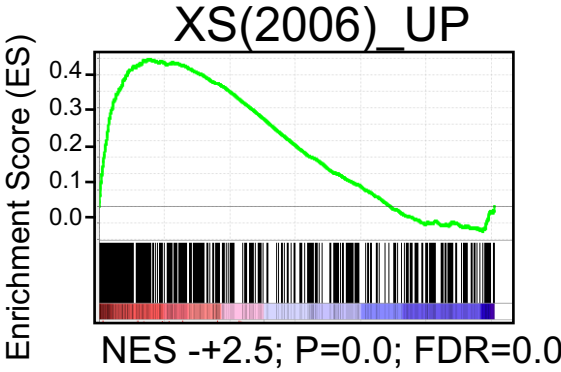

*rhb1/rhb1*

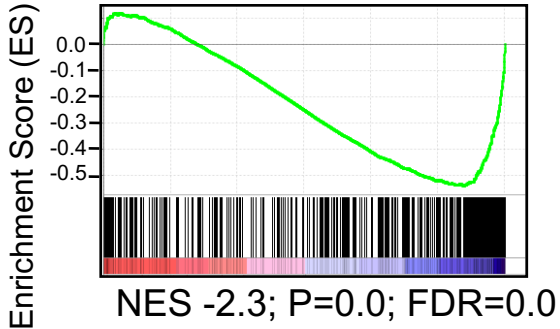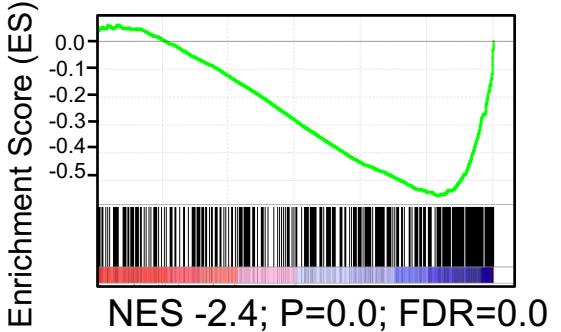

(b)

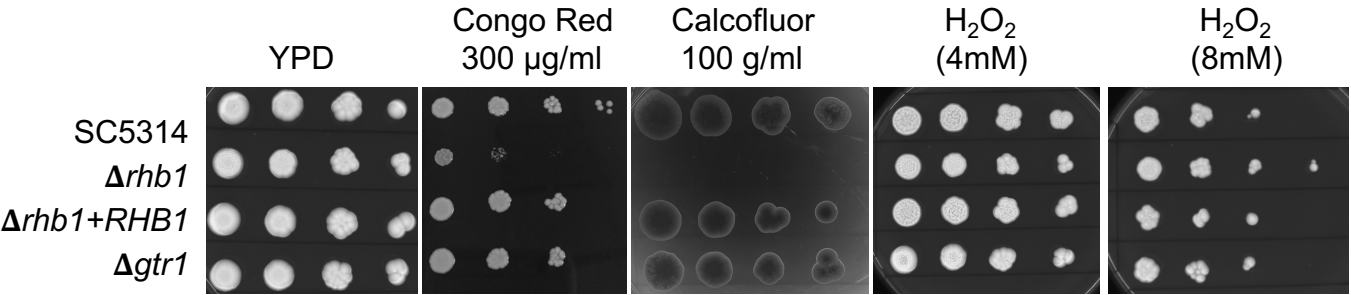

Supplement: FIG S4 [file sph006172401sf8.pdf]
