# Supplementary material for: The Candida albicans TOR-Activating GTPases Gtr1 and Rhb1 Coregulate Starvation Responses and Biofilm Formation
Source: mSphere. 2017 Nov 15;2(6):e00477-17. doi: 10.1128/mSphere.00477-17 (PMC5687921; doi:10.1128/mSphere.00477-17)
Supplement: TABLE S1 [file sph006172401st1.docx]

| **Strain** | **Parent** | **Genotype** | **Reference** |
| --- | --- | --- | --- |
| SC5314 | N/A | Wild Type | (1) |
| P*_ENO_VAM6* | SC5314 | *CaNAT1*::*ENO1p*::*VAM6* | This study |
| P*_ENO_TOR1* | SC5314 | *CaNAT1*::*ENO1p*::*TOR1* | This study |
| P*_ENO_RHB1* | SC5314 | *CaNAT1*::*ENO1p*::*RHB1* | This study |
| P*_ENO_GTR1* | SC5314 | *CaNAT1*::*ENO1p*::*GTR1* | This study |
| ∆*gtr1* | SC5314 | *gtr1*::FRT/*GTR1* | This study |
| ∆∆*gtr1* | ∆*gtr1* | *gtr1*::FRT/*gtr1*::*SAT1* | This study |
| *gtr1/GTR1* | ∆∆*gtr1* | *gtr1*::FRT/*gtr1*::*SAT1*; *cdr1*::pCDRI-*GTR1*/*CDR1* | This study |
| CCT-D1 | SC5314 | *rhb1*::FRT/*rhb1*::FRT | (2) |
| CCT-RB1 | CCT-D1 | *rhb1*::FRT/*rhb1*::*RHB1-SAT1* | (2) |

1. **Gillum AM**, **Tsay EY**, **Kirsch DR**. 1984. Isolation of the *Candida albicans* gene for orotidine-5'-phosphate decarboxylase by complementation of *S. cerevisiae ura3* and *E. coli* pyrF mutations. Mol Gen Genet **198**:179–182.

2. **Tsao C-C**, **Chen Y-T**, **Lan C-Y**. 2009. A small G protein Rhb1 and a GTPase-activating protein Tsc2 involved in nitrogen starvation-induced morphogenesis and cell wall integrity of *Candida albicans*. Fungal Genetics and Biology **46**:126–136.
