# Supplementary material for: The Candida albicans TOR-Activating GTPases Gtr1 and Rhb1 Coregulate Starvation Responses and Biofilm Formation
Source: mSphere. 2017 Nov 15;2(6):e00477-17. doi: 10.1128/mSphere.00477-17 (PMC5687921; doi:10.1128/mSphere.00477-17)
Supplement: TABLE S2 [file sph006172401st2.docx]

| Transformation Oligonucleotide | | Oligonucleotide Sequence |
| --- | --- | --- |
| ENOp/TOR Mut F1 | TTTTTTTCAACCCCGAAATTTGTTCTTCTGATTTTTTGACTCCATTACCTACCGTTGTGGACCAAGTGATTCGTTAGTATCGAATCGACAGC | |
| ENOp/TOR Mut R1 | TCCTTTGAAAATCTCTGTTAGAGCAACACCATCAACCACTTGTAGTTGTGCTGATGTAGATGCTGATGCTGACATTGTTGTAATATTCCTGAATTATC | |
| ENOp/RHB1 Mut F1 | TTTGATAACCACCACCACCAAGTGAAGCAAGCAAGCAGCCAAACAAAAAAAAATTTTCTATTATTTTATTGTTGTCCTTTTTTTTTTCGTTAGTATCGAATCGACAGC | |
| ENOp/RHB1 Mut R1 | TCTTCAACAAATCGAACGGTGATGGAGGATTTTCCAACTGAACGAGATCCAACCACTGCTATCTTACGGGCTTTGACCAGCATTGTTGTAATATTCCTGAATTATC | |
| ENOp/GTR1 Mut F1 | AAGTATAGTCATATCATATTTATAATTCTACAAATAAGGTCAAAAAATAAATATAAAAAACCAAAAATACGAACACCTAACGTTAGTATCGAATCGACAGC | |
| ENOp/GTR1 Mut R1 | AAGTATAGTCATATCATATTTATAATTCTACAAATAAGGTCAAAAAATAAATATAAAAAACCAAAAATACGAACACCTAACGTTAGTATCGAATCGACAGC | |
| GTR1_F1 | CAAAATGTCAATCTCTCGTGTATAACATTTACACTAATTTATACTCAATCTTAACTATCATCATCATCATTAACAACAACAGTAAAACGACGGCCAGT | |
| GTR_R1 | ATAGAAACTGATTCGTGATTTTGTTAATAGTTTTAATTACAGATTAAAACTTAAAACTCCCACCTGTTAAGTTTGACTCTGGAAACAGCTATGACCATG | |
| GTR 2ND allele F1 | TAATCATAATCATTTCTATTTAAATTTAAATTATTGAAAACAAAACAATCTCACTTTTTTCATCTTATAACTAGTAAAACGACGGCCAGT | |
| GTR1 2ND allele R1 | TGTTTTACTCATTATTTTCAATTTTTTCAAACCATCTTCTAGCAGCTTTAATATTATCTAAAATCAATAATTCTTCATGTGGAAACAGCTATGACCATG | |
| GTR1_Reint F1 | CCGCTCGAGGAATGTATACCACTTCTATG | |
| GTR1_Reint R1 | GGGAAGCTTACGCGCGTGACACTCTTCGG | |
| GTR1_Flank1 | ATTGATGAACGAGATGGATGGG | |
| GTR1_Flank1 | GACTCTACCAGTCTAAGTA | |
| GTR1_IntF | TCAGCATTAGATACTCGACG | |
| GTR1_IntF | TGAACAAACAATTTGTGACC | |
| ENOp Con F1 | TTGATAATTCAGGAATATTACAAC | |
| TOR1 Con R1 | TTTGAAGCCATTCAATAGCTCTTTT | |
| RHB1 Con R1 | TCATTTTTGTTACCAACAAG | |
| GTR1 Con R1 | TAATGCTGAATAATTGGAAA | |
| GTR1/SAT1 FLIP F1 | ATTATTGTTTATTGTAAGAT | |
| GTR1/SAT1 FLIP R1 | ATTGGGCTGGCAGTAATTTC | |
| ACT1 F1 | AGCTCCAGAAGCTTTGTTCAGACCAG | |
| ACT1 R1 | TGCATACGTTCAGCAATACCTGGG | |
| MEP2 F1 | GGAAGCAATTGGGGTATCAG | |
| MEP2 R1 | TGGTTTCAGGATCGTCATCA | |
